# Combined Vildagliptin and Metformin Exert Better Cardioprotection than Monotherapy against Ischemia-Reperfusion Injury in Obese-Insulin Resistant Rats

**DOI:** 10.1371/journal.pone.0102374

**Published:** 2014-07-18

**Authors:** Nattayaporn Apaijai, Kroekkiat Chinda, Siripong Palee, Siriporn Chattipakorn, Nipon Chattipakorn

**Affiliations:** 1 Cardiac Electrophysiology Research and Training Center, Faculty of Medicine, Chiang Mai University, Chiang Mai, Thailand; 2 Cardiac Electrophysiology Unit, Department of Physiology, Faculty of Medicine, Chiang Mai University, Chiang Mai, Thailand; 3 Department of Oral Biology and Diagnostic Sciences, Faculty of Dentistry, Chiang Mai University, Chiang Mai, Thailand; 4 Biomedical Engineering Center, Chiang Mai University, Chiang Mai, Thailand; 5 School of Medicine, Mae FahLuang University, Chiang Rai, Thailand; University of Hull, United Kingdom

## Abstract

**Background:**

Obese-insulin resistance caused by long-term high-fat diet (HFD) consumption is associated with left ventricular (LV) dysfunction and increased risk of myocardial infarction. Metformin and vildagliptin have been shown to exert cardioprotective effects. However, the effect of these drugs on the hearts under obese-insulin resistance with ischemia-reperfusion (I/R) injury is unclear. We hypothesized that combined vildagliptin and metformin provide better protective effects against I/R injury than monotherapy in obese-insulin resistant rats.

**Methodology:**

Male Wistar rats were fed either HFD or normal diet. Rats in each diet group were divided into 4 subgroups to receive vildagliptin, metformin, combined vildagliptin and metformin, or saline for 21 days. Ischemia due to left anterior descending artery ligation was allowed for 30-min, followed by 120-min reperfusion. Metabolic parameters, heart rate variability (HRV), LV function, infarct size, mitochondrial function, calcium transient, Bax and Bcl-2, and Connexin 43 (Cx43) were determined. Rats developed insulin resistance after 12 weeks of HFD consumption. Vildagliptin, metformin, and combined drugs improved metabolic parameters, HRV, and LV function. During I/R, all treatments improved LV function, reduced infarct size and Bax, increased Bcl-2, and improved mitochondrial function in HFD rats. However, only combined drugs delayed the time to the first VT/VF onset, reduced arrhythmia score and mortality rate, and increased p-Cx43 in HFD rats.

**Conclusion:**

Although both vildagliptin and metformin improved insulin resistance and attenuate myocardial injury caused by I/R, combined drugs provided better outcomes than single therapy by reducing arrhythmia score and mortality rate.

## Introduction

Long-term high-fat diet consumption is known to cause obesity, insulin resistance, and cardiac dysfunction [1], and have been shown to increase the risk of having ischemic heart disease [2]. While revascularization is the goal of myocardial ischemia treatment, reperfusion therapy is known to induce reperfusion injury and lead to greater myocardial damage. This adverse effect has been shown to be even more severe in obese insulin resistance, resulting in increased infarct size and increased myocardial susceptibility to ischemia-reperfusion (I/R) injury [3], [4]. I/R injury has been shown to associate with fatal cardiac arrhythmias [5]. Cardiac mitochondrial damage has been implicated as one of the major factors responsible for myocardial cell death from I/R injury [6]. Several lines of evidence showed that increased intracellular and mitochondrial calcium accumulation can trigger cell apoptosis [6], [7]. Previous studies also demonstrated that decreased Connexin 43 (Cx43), the principal gap junction protein, is found in acute myocardial infarction [8]. Many studies demonstrated that loss of Cx43expression in the heart could result in a markedly increase in incidence of arrhythmia [9], [10].

Several anti-diabetic drugs including dipeptidyl peptidase 4 (DPP-4) inhibitors and metformin have been shown to exert cardioprotective effects in addition to their glycemic control [11], [12]. A recent study reported the beneficial effects of combined DPP-4 inhibitor and metformin on metabolic parameters in type 2 diabetic patients [13]. However, the role of these drugs including combined drugs therapy on cardiac function, heart rate variability (HRV), cardiac mitochondrial function, and intracellular calcium regulation during I/R in long-term HFD-induced obese insulin resistant rats have not been investigated.

We have previously reported the protective effects of vildagliptin and metformin on metabolic parameters and LV function in obese-insulin resistant rats [14]. In the present study, we determine the effects of a DPP-4 inhibitor vildagliptin, metformin, and combined drugs on the hearts of obese-insulin resistant rats underwent I/R injury. We hypothesized that vildagliptin and metformin provide cardioprotective effects against I/R injury in obese-insulin resistant rats, and we demonstrate that combined drugs exert better cardioprotection than either single drug regimen.

## Materials and Methods

### Animals and diet

All experiments in this study were approved by the Faculty of Medicine, Chiang Mai University Institutional Animal Care and Use Committee (Permit No. 29/2555), in compliance with NIH guidelines, and in accordance with the ARRIVE guidelines for reporting experiments involving animals [15]. Male Wistar rats weighing 180–200 g were obtained from the National Animal Center, Salaya Campus, Mahidol University, Bangkok, Thailand. Rats were allowed to acclimate for seven days and housed in 12/12 hour dark/light cycle with controlled temperature (25°C). They were divided into 2 groups to receive either normal diet (ND; a standard laboratory pelleted diet containing 19.8% energy from fat) or high-fat diet (HFD; a diet containing 59.3% energy from fat) [1], [16]. Rats were fed with their diet for 15 weeks. At week 12, rats in each dietary group were divided into 4 subgroups to receive vildagliptin 3-mg/kg/day (Galvus, Novartis, Bangkok, Thailand) [14], metformin 30-mg/kg/day (Glucophage, Merkserono, Bangkok, Thailand) [14], combination of vildagliptin and metformin, or vehicle (0.9% normal saline solution in an equal volume) for another 21 days via gavage feeding. Body weight and food intake were recorded weekly, and blood samples were collected from the tail vein at baseline, 12^th^ week, and at the end of treatment (15^th^ week). HRV was determined at baseline, 4^th^ week, 8^th^week, 12^th^week, and 15^th^week (the end of pharmacological interventions).

### Metabolic parameters

Plasma glucose, total cholesterol, and triglyceride levels were determined using a commercial colorimetric assay kit (Erba Diagnostics Mannheim GmbH, Mannheim, Germany) [16]. Plasma HDL level was determined using a commercial colorimetric assay kit (Biovision, California, USA) [1], and plasma LDL level was calculated using Friedewald equation [17]. Plasma insulin level was determined using a commercial sandwich ELISA kit (LINCO research, Missouri, USA) [1]. The Homeostasis Model Assesment (HOMA) index was used to assess insulin resistance. Increased HOMA index indicates a higher degree of insulin resistance [18].

### Plasma and cardiac malondialdehyde (MDA) level

Plasma and cardiac MDA level was determined using high performance liquid chromatography (HPLC) based assay (Thermo scientific, Bangkok, Thailand) [1]. Plasma was mixed with H_3_PO_4_ and thiobarbituric acid (TBA) to produce thiobarbituric acid reactive substances (TBARS). Plasma TBARS was determined from a standard curve and reported as equivalent to the MDA concentration [1].

### Heart rate variability (HRV)

Lead II electrocardiogram (ECG) was recorded for 20 minutes in conscious rats using PowerLab (AD instruments, Sydney, Australia) with Chart 5.0 program. Power spectra of R-R interval variability were obtained using Fast Fourier Transform (FFT) algorithm [1], [19]. High-frequency band (HF; 0.6–3 Hz), Low-frequency band (LF; 0.2–0.6 Hz), and Very low-frequency band (VLF; below 0.2 Hz) were detected and calculated as integrals under the respective part of the power spectral density function. To minimize the effect of changes in total power on the LF and HF bands, LF and HF bands were expressed as normalized units by dividing them with the total power minus VLF [19]. LF/HF ratio was analyzed using analytical program [1]. The LF/HF ratio was considered a measure of cardiac sympathovagal balance. Increased LF/HF ratio indicated depressed HRV [19].

### Echocardiography

At baseline, 4^th^week, 8^th^week, 12^th^week, and 15^th^ week (post-treatment), echocardiographic parameters were measured in each rat using a HP/Agilent Philips Sonos 4500 (Agilent technologies, Massachusetts, USA). An echocardiography probe was placed in gentle contact with the chest, and images were collected along the parasternal short axis of the heart [20]. M-mode echocardiography was performed at the level of the papillary muscles. %Fractional shortening (%FS) and left ventricular ejection fraction (LVEF) were determined.

### Ischemia-reperfusion injury protocol

**Figure 1:**
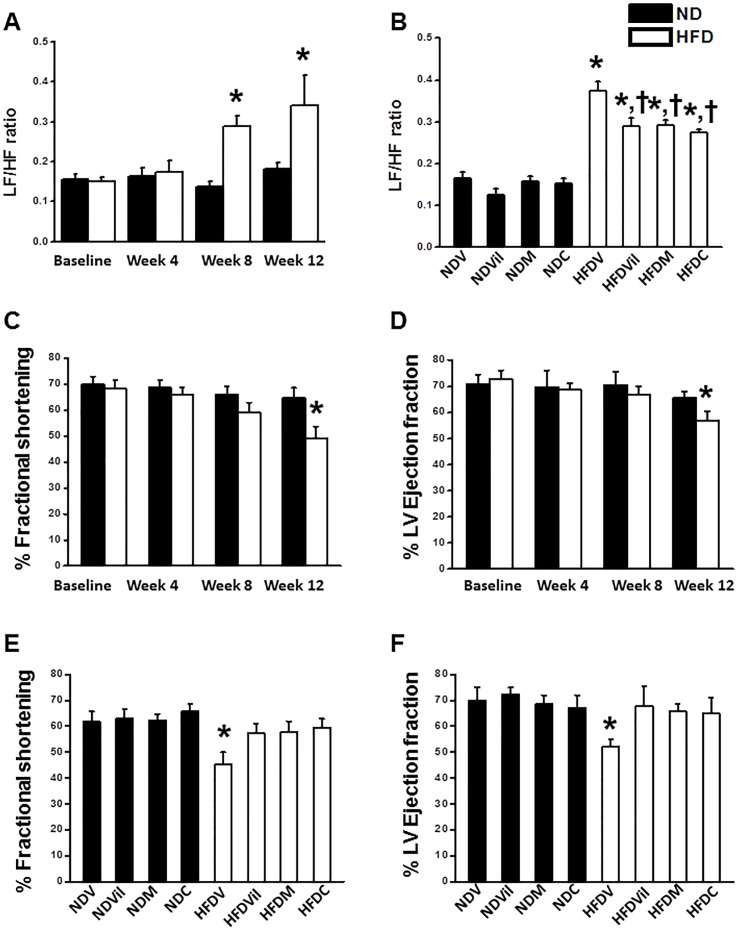
Effects of HFD consumption, vildagliptin, metformin, and combined drugs on HRV and echocardiographic parameters. (A) The LF/HF ratio was increased at week 8^th^ and week 12^th^of HFD consumption.*p<0.05 vs. baseline, n = 11/group. (B) Vildagliptin, metformin, and combined drugs reduced the LF/HF ratio in HFD rats. *p<0.05 vs. NDV, ^†^p<0.05 vs. HFDV, n = 11/group, (C) %FS was decreased after 12 weeks of HFD consumption.*p<0.05 vs. ND, n = 11/group. (D) LVEF was decreased after 12 weeks of HFD consumption.*p<0.05 vs. ND, n = 11/group. (E) Vildagliptin, metformin, and combined drugs increased %FS in HFD rats. *p<0.05 vs. HFDV, n = 11/group. (F) Vildagliptin, metformin, and combined drugs increased LVEF in HFD rats. *p<0.05 vs. HFDV, n = 11/group.

At the end of treatment, rats were subject to I/R injury protocol (Figure 1). Rats were anesthetized using Zoletil (50 mg/kg, Virbac Laboratories, Carros, France) and Xylazine (0.15 mg/kg, LaboratoriosCalier, Barcelona, Spain), intramuscularly. Zoletil is a combination of a dissociative anesthetic agent, tiletamine hypochloride, and a tranquilizer, zolazepam hydrochloride. The onset of action is 3–5 minutes by intramuscular route. We checked the depth of anesthesia by testing the absence of eyelid reflex and the tail pinch reflex. To maintain anesthesia, we used xylazine (0.15 mg/kg) which is an 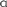
2 adrenergic receptor agonist results in sedation, analgesia, and muscle relaxation and provides a sedative effect within 1–3 hours. Tracheostomy was performed and rats were ventilated with room air from a positive pressure rodent ventilator (CWE, Inc, Ardmore, Pennsylvania, USA). Lead II ECG was recorded throughout the experiment to determine the time to the 1^st^ ventricular tachycardia/ventricular fibrillation (VT/VF) onset, VT/VF incidence, and arrhythmia scores. Arrhythmia score was characterized in accordance with the Lambeth conventions and using a score as described by Curtis and Walker [21]. A left-side thoracotomy was performed at the fourth intercostal space, and the pericardium was incised to expose the heart. Left anterior descending (LAD) coronary artery was identified and ligated at approximately 2 mm distal to its origin [5], [22]. The end of a ligature was passed through a small vinyl tube, which is used to occlude the LAD by pulling the thread. ST elevation was used to confirm ischemia. The heart was subject to ischemia for 30 minutes, then the ligature was loosened for the ischemic myocardium to be reperfused for 120 minutes.

### Left-ventricular (LV) function

The right carotid artery was identified and the pressure-volume (P-V) catheter (Scisense, Ontario, Canada) was inserted and advanced into the left ventricle to determine the cardiac function during I/R injury. Heart rate (HR), end-systolic pressure (ESP), end-diastolic pressure (EDP), maximum pressure (Pmax), minimum pressure (Pmin), dP/dtmax, dP/dtmin, stroke work (SW), and stroke volume/body weight (SV/BW) were determined prior to I/R injury protocol, at the end of occlusion, and at the end of reperfusion using analytical software program (Labscribe, Dover, New Hampshire, USA).

### Infarct size determination

After 150 minutes of I/R period, the heart was excised and mount on the modified Langendorff apparatus via the aorta. Cold saline solution was used to flush out the blood, after which the LAD was re-occluded and 1-ml of 0.5% (w/v) Evans blue dye was injected to define the area at risk. Evans blue dye stained the perfused myocardium, whereas the LAD occlusion area did not stained with Evans blue dye, after which the heart was sliced into pieces with approximately 1-mm thickness for each slice and incubated with 1% buffered 2,3,5-triphenyltetrazolium chloride (TTC) (Sigma-Aldrich, St. Louis, Missouri, USA), followed by placing them in 10% formalin overnight [5], [22]. The infarct and remote areas were determined using the image tool software version 3.0. The infarct size was calculated depending on the weight of each slice according to Reiss et al.'s formula [5], [11], [23].

### Determination of cardiac mitochondrial function

For mitochondrial study, mitochondria were obtained from the ischemic myocardium and remote myocardium from fresh hearts. Ischemic area was obtained from left ventricular apex and remote area was obtained from right ventricle. The heart was minced and homogenized in the mitochondrial isolation buffer, pH 7.2, containing 300 mM sucrose, 0.2 mM EGTA, 5 mM TES. Homogenates was centrifuged at 800 g, 4°C for 5 min. The supernatant was collected and centrifuged at 8800 g, 4°C for 5 min. Then, the mitochondrial pellet was resuspended in the mitochondrial isolation buffer and centrifuged at 8800 g, 4°C for 5 min. The mitochondria were collected and the protein concentration was determined by the bicinchoninic acid method [22]. Cardiac mitochondrial function was assessed by determining cardiac mitochondrial reactive oxygen species (ROS) production, cardiac mitochondrial membrane potential change, and cardiac mitochondrial swelling [11], [14], [24].

#### Cardiac mitochondrial ROS production

Cardiac mitochondria were incubated with2 µM DCFH-DA dye at 25°C for 20 minutes. The dye was excited at λ_ex_ 485 nm and detected at λ_em_ 530 nm using fluorescent microplate reader (BioTek Instruments, Winooski, Vermont, USA). An increase in the fluorescent intensity indicated an increased mitochondrial ROS production [1], [11], [24].

#### Cardiac mitochondrial membrane potential changes

Cardiac mitochondrial membrane potential changes were determined using 5- µM JC-1 dye as described previously [1], [24]. In brief, cardiac mitochondria were incubated with JC-1 at 37°C for 30 minutes. JC-1 monomer form (green fluorescent) was excited at λ_ex_ 485 nm and detected at λ_em_ 590 nm, and JC-1 aggregate form (red fluorescent) was excited at λ_ex_ 485 nm and detected at λ_em_ 530 nm using a fluorescent microplate reader. An increase in the red/green fluorescent intensity ratio indicates cardiac mitochondrial membrane depolarization [1], [11], [24].

#### Cardiac mitochondrial swelling

Cardiac mitochondria were incubated with 1.5-mM respiration buffer containing 100 mMKCl, 10 mM HEPES, 5 mM KH2PO4, and the absorbance was determined using a spectrophotometer as described previously [1], [24]. Cardiac mitochondrial swelling is indicated by a decrease in the detected absorbance [1], [11], [24].

#### Cardiac mitochondria morphology

Cardiac mitochondria were fixed with 2.5% glutaraldehyde in a 0.1-M phosphate buffer overnight and post fixed in a 1% cacodylate-buffer osmium tetroxide for 2 hours, then dehydrated with graded series of ethanol [24]. Cardiac mitochondria were embedded in Epon-Araldite, cut with a diamond knife, and stained with uranyl acetate and lead acetate[24]. Cardiac mitochondria morphology were detected using a transmission electron microscope [24].

### Western blot analysis

The myocardial tissues for western blot study was obtained from the fresh heart at the end of the I/R protocol. The ischemic myocardium was obtained from the left ventricular apex and intact myocardium was obtained from the right ventricle. Myocardial protein extracts were prepared by a homogenization of nitrogen-frozen myocardial tissues (ischemia and remote area) in a 300- µl extraction buffer containing 20-mMTris-HCl (pH 6.8), 1-mM Sodium orthovanadate, 5-mMSodium fluoride, and protease inhibitor. Total protein concentration was determined using a Bio-Rad protein assay kit (Bio-Rad Laboratories, Hercules, California, USA). Fifty to 80 µg of total protein were mixed with loading buffer (5% betamercaptoethanol, 0.05% bromophenol blue, 75 mMTris-HCl (pH 6.8), 2% SDS and 10% glycerol), and loaded onto the 10% SDS-Acrylamide gels. Proteins were transferred to polyvinylidenedifluoride (PVDF) membrane in a glycine/methanol transferred buffer [5], [22] in a Wet/Tank blotting system (Bio-Rad Laboratories, Hercules, California, USA). Membranes were blocked in 5% skim milk in Tris-Buffered Saline and Tween (TBST) buffer. Western blot analysis for Bax, Bcl-2, total Connexin-43, and Connexin43 phosphorylated at Ser368 was done in myocardial tissues. Membranes were exposed to mouse polyclonal anti-rat Bax and Bcl-2 (1∶1000 dilution, Santa Cruz Biotechnology, Dallas, Texas, USA), Phospho-Cx43(Ser368), Cx43 (1∶1000 dilution, Cell Signaling Technology, Danvers, Massachusetts, USA). Bound antibody was detected by horseradish peroxidase conjugated with anti-rabbit IgG (1∶2000 dilution, Cell Signaling Technology, Danvers, Massachusetts, USA). The membranes were developed using the Amersham enhanced chemiluminescence Prime Western Blotting Detection Reagent (GE healthcare, Buckinghamshire, UK) and densitometric analysis was carried out [5].

### Ventricular cardiomyocyte isolation

A separated group of thirty-two male Wistar rats was used in the intracellular calcium transient study. Rats were anesthetized by intraperitoneal injection of thiopental (1 ml/kg, Research institute of antibiotics and biotransformation, Prague, Czechoslovakia), and 0.2-ml heparin (LEO pharma, Ballerup, Denmark) was injected into the femoral vein. The heart was excised quickly with decent length of aorta and mounted on the cannula and tied with suture. The blood was washed out with solution 1 (Tyrode's solution and 750 µM CaCl_2_) then switched to solution 2 (Tyrode's solution and 100 µM ethylene glycol tetraacetic acid (EGTA) for 4 minutes, and switched to solution 3 (Tyrode's solution, 100 µM CaCl_2_, and 1 mg/ml collagenase (Worthington, Lakewood, New Jersey, USA)) for 10 minutes. The ventricles were cut and triturated in solution 3 at 37°C for 30 minutes, and were then filtered with a nylon mesh. The cells were left to settle down for 10 minutes and solution 3 was removed. Cells were resuspened in solution 4 (Tyrode's solution, 0.5 mM CaCl_2_, and 1% BSA) for 10 minutes. Cells were resuspened in 10 ml of solution 5 (Tyrode's solution, 1 mM CaCl_2_) and were ready for calcium transient study [22].

### Intracellular calcium transient

The measurement of cardiomyocyte calcium transient was performed using a modified method from Wongchareon*et al*. [25] as described previously [22]. Isolated cardiomyocytes were incubated with 25- µM Fura-2/AM (Sigma Chemical, St. Louis, Massachusetts, USA) in 37°C, 5% CO_2_, for 30 minutes. Fura-2 was excited at ultraviolet light wavelength of 340 nm and 387 nm by monochromator from xenon arc lamp controlled by a microfluorometry system (OSP 100-CA, Olympus, Tokyo, Japan). The excitation light was directed into an inverted microscope (IX-70, Olympus, Tokyo, Japan). The emitted fluorescent intensity from Fura-2 was detected at wavelength 500 nm and digitized at 200 Hz. The ratio of fluorescent emission of two wavelengths was recorded as the index of intracellular calcium. The intracellular calcium transient peak amplitude and decay rate were measured during 1-Hz field-stimulation with a 10-ms twice-threshold strength square-pulse wave [22]. After baseline recording, 2-mM hydrogen peroxide (H_2_O_2_) was applied onto cardiomyocytes to induce oxidative stress (mimicking the ischemic process), after which the ratio of fluorescent emission of two wavelengths was recorded to determine the differences on intracellular calcium transient under oxidative stress condition among cardiomyocytes taken from the hearts of rats treated with vildagliptin, metformin, combined drugs, and vehicle.

### Statistical Analysis

Data were expressed as mean ± SEM. One-way ANOVA followed by Fisher's least significant difference post hoc was used to test the different among the group. Mortality rate and VT/VF incidence were analyzed using Chi-square test. Mitochondrial function was analyzed using Kruskal-Wallis H followed by Mann-Whitney U test. *P*<0.05 was considered statistical significant.

## Results

### Effects of metformin and vildagliptin on metabolic parameters

At baseline, metabolic parameters were not different between ND and HFD group (Table 1). After 12 weeks of HFD consumption, the body weight, plasma insulin, plasma cholesterol, plasma MDA levels and HOMA index were increased in HFD rats, compared with ND rats (Table 1). Vildagliptin, metformin, and combined drugs reduced plasma insulin, plasma total cholesterol, plasma LDL, plasma and cardiac MDA levels and increased plasma HDL level in HFD rats, compared with HFD rats treated with vehicle (HFDV) (Table 2). No difference on these parameters was found between monotherapy and combined drugs.

**Table 1:** Effects of HFD consumption on metabolic parameters at baseline and 12-week after HFD consumption.

Metabolic parameters	Baseline		Week 12	
	ND	HFD	ND	HFD
Body weight (g)	191±2	190±9	480±10*	595±28*,^†^
Food intake (g/day)	26±1	29±1	29±2	26±2
Plasma insulin (ng/ml)	1.8±0.2	1.7±0.3	1.6±0.5	4.4±0.7*,^†^
Plasma glucose (mg/dl)	130±9	135±6	131±6	137±8
HOMA index	15±2	14±3	14±4	35±9*,^†^
Plasma total cholesterol (mg/dl)	41±5	42±4	41±2	53±3*,^†^
Plasma triglyceride (mg/dl)	70±6	72±8	80±8	87±5
Plasma HDL (mg/dl)	9.6±1	8.3±0.8	10.7±1	7.4±1.7*,^†^
Plasma LDL (mg/dl)	17.4±1	19.4±0.8	14.3±1.3	28.2±1.7*,^†^
Plasma MDA (µmol/ml)	2.9±0.2	3.1±0.1	3.3±0.2	6.4±0.1*,^†^

ND  =  normal diet, HFD  =  high-fat diet. *P<0.05 vs. Baseline, ^†^ *P*<0.05 vs. ND week 12.

**Table 2:** Effects of vildagliptin, metformin, and combination of vildagliptin and metformin on metabolic parameters (n = 11/group).

Parameters	NDV	NDVil	NDM	NDC	HFDV	HFDVil	HFDM	HFDC
Body weight (g)	478±11	454±14	439±12	472±14	608±18*	567±28*	566±7*	586±36*
Visceral fat (g)	22±1	23±2	22±3	22±1	50±4*	42±4*	46±3*	56±5*
Food intake (g/day)	22±1	25±1	22±3	23±2	22±2	25±1	19±2	19±1
Plasma glucose (mg/dl)	135±12	143±12	130±14	139±13	138±9	132±5	134±16	135±11
Plasma insulin (ng/ml)	2.3±0.3	1.7±0.4	2.1±0.4	2.3±0.3	5.4±0.6*	3.1±0.5^†^	3.8±0.2^†^	3.3±0.2^†^
HOMA index	19±3	18±5	16±4	20±3	32±5*	23±4^†^	26±2^†^	22±1^†^
Plasma total cholesterol (mg/dl)	49±6	44±4	49±2	54±3	63±4*	43±3^†^	51±2^†^	42±3^†^
Plasma triglyceride (mg/dl)	86±5	79±1	85±4	86±2	88±4	80±2	87±4	84±2
Plasma HDL (mg/dl)	12.1±1	10.9±2	11.8±2	10±1	7.6±1*	10.6±2^†^	10.9±1^†^	12±2^†^
Plasma LDL (mg/dl)	19.7±1	17.2±2	20.2±2	20±3	37±1*	20.4±2^†^	22.7±1^†^	23.2±1^†^
Plasma MDA (µmol/ml)	3.6±0.1	3.5±0.1	3.7±0.1	3.1±0.1	6.8±0.1*	6.1±0.2*,^†^	6.3±0.2*,^†^	6.3±0.1*,^†^
Cardiac MDA (µmol/mgprotein)	23.5±3.4	12.8±3.9*	14.5±2.8*	11.8±2.9*	32.7±4.6*	12.8±1.6^†^	14.2±1.7^†^	12.5±2.7^†^

*P<0.05 vs. NDV, ^†^ *P*<0.05 vs. HFDV.

### Effects of metformin and vildagliptin on HRV

At baseline, the LF/HF ratio was not different between ND and HFD rats (Figure 1A). The LF/HF ratio was increased after 8 weeks of HFD consumption (Figure 1A). After treatment, the LF/HF ratio was not different in ND groups (Figure 1B). Treatments with vildagliptin, metformin, and combined drugs significantly reduced the LF/HF ratio in HFD rats (Figure 1B), indicating an improved cardiac sympathovagal balance in HFD rats.

### Effects of metformin and vildagliptin on left ventricular function prior to I/R

At baseline, %FS and LVEF were not different between ND and HFD. We found that %FS and LVEF were decreased after 12 weeks of HFD consumption, compared with ND (Figure 1C–D). After treatment, vildagliptin, metformin, and combined drugs restored %FS and LVEF in HFD rats, compared with HFDV (Figure 1E–F).

### Effects of metformin and vildagliptin on arrhythmia score during I/R

**Figure 2:**
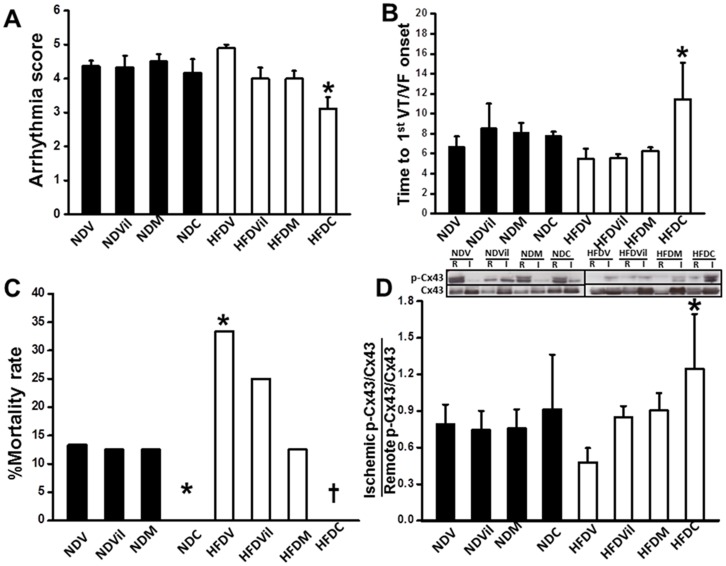
Effects of vildagliptin, metformin, combined drugs on arrhythmia, mortality rate, and phosphorylation of Cx43 level. (A) Combined drugs reduced arrhythmia score in HFD rats.*p<0.05 vs. HFDV, n = 11/group. (B) Combined drugs delayed time to the 1^st^ VT/VF onset in HFD rats. *p<0.05 vs. HFDV, n = 11/group. (C) Mortality rate was higher in HFDV, and combined drugs reduced mortality rate in both ND and HFD rats. *p<0.05 vs. NDV, ^†^p<0.05 vs. HFDV, n = 11/group. (D) Combined drugs increased p-Cx43 in HFD rats. *p<0.05 vs. HFDV, n = 5/group.

Arrhythmia score tended to be higher during I/R in HFDV than NDV rats, but the value did not reach statistical significance (Figure 2A). Only HFD rats treated with combined drugs had reduced arrhythmia score (Figure 2A). The time to 1^st^ VT/VF onset was not different between NDV and HFDV. In ND rats, time to 1^st^ VT/VF onset was not different among different treatment groups. In HFD rats, only combined drugs delayed time to the first VT/VF onset in HFD rats (Figure 2B). We also found that HFDV rats had a higher mortality rate, compared with the NDV group (Figure 2C). The mortality rate was reduced only in ND and HFD rats treated with combined drugs (Figure 2C). We determined the levels of Cx43 phosphorylation in heart tissues since it has been shown to closely associate with arrhythmia during I/R, and found that Cx43 phosphorylation was not different among the different treatments in ND groups (Figure 2D). However, in HFD rats, the phosphorylation of Cx43 was increased only in HFD rats treated with combined drugs (Figure 2D).

### Effects of metformin and vildagliptin on left ventricular function during I/R

Baseline LV function data by P-V loop assessment prior to I/R are shown in Table 3. LV function was not different among ND groups prior to I/R injury. ESP, SV/BW were decreased and EDP was increased in HFDV rats, compared with NDV group (p<0.05 vs. NDV, Table 3). All treatments increased ESP and SV/BW and reduced EDP in HFD rats (p<0.05 vs. HFDV, Table 3).

**Table 3:** Effects of metformin, vildagliptin, and combination of vildagliptin and metformin on cardiac function before I/R injury (n = 11/group).

Cardiac function	NDV	NDVil	NDM	NDC	HFDV	HFDVil	HFDM	HFDC
Heart rate (bpm)	237±15	199±17	221±12	213±28	241±18	244±11	209±7	230±15
ESP (mmHg)	85±6	83±9	81±3	94±7	68±5*	93±10^†^	90±10^†^	91±9^†^
EDP (mmHg)	10±2	9±3	6±1	9±5	21±6*	8±2^†^	8±2^†^	10±1^†^
Pmax (mmHg)	108±7	87±9	82±11	101±5	93±8	117±15	94±11	114±14
Pmin (mmHg)	11±5	5±3	3±1	8±3	14±4	9±3	8±2	10±2
dP/dtmax (mmHg/s)	10090±1501	9487±442	10189±369	10561±781	8439±393	10081±1945	9077±737	11626±1974
dP/dtmin (mmHg/s)	−6378±1281	−4557±1162	−4119±891	−6205±454	−5388±428	−5226±800	−4714±632	−5761±1292
SW (mmHg/ml)	14653±784	12541±1440	14528±1106	14813±935	13698±1433	14929±1893	13495±1568	15446±1268
SV/BW (µl)	1.06±0.12	1.06±0.13	1.12±0.10	1.16±0.15	0.65±0.05*	1.08±0.14^†^	1.10±0.09^†^	1.04±0.2^†^

*P<0.05 vs. NDV, ^†^ *P*<0.05 vs. HFDV.

LV function data at the end of LAD occlusion are shown in Table 4. At the end of LAD occlusion, all treatments increased the dP/dt_max_, and combined drugs also increased the ESP in ND rats, compared with the NDV group (p<0.05 vs. NDV). In HFDV rats, the HR and EDP were increased and the ESP and SV/BW was decreased (p<0.05 vs. NDV). All treatments reduced the EDP, and increased the ESP, dP/dt_max_, and SV/BW in HFD rats (p<0.05 vs. HFDV, Table 4).

**Table 4:** Effects of vildagliptin, metformin, and combination of vildagliptin and metformin on cardiac function at the end of Occlusion (n = 11/group).

Cardiac function	NDV	NDVil	NDM	NDC	HFDV	HFDVil	HFDM	HFDC
Heart rate (bpm)	214±11	200±12	227±17	213±12	266±23*	249±14	243±23	242±26
ESP (mmHg)	74±4	82±7	83±20	103±3*	66±7*	90±8^†^	93±6^†^	90±7^†^
EDP (mmHg)	15±4	10±1	11±2	9±2	26±4*	13±2^†^	12±1^†^	14±2^†^
Pmax (mmHg)	95±5	96±9	99±20	105±3	106±11	94±8	102±6	109±10
Pmin (mmHg)	10±4	5±1	6±1	12±2	11±3	9±3	13±2	14±2
dP/dtmax (mmHg/s)	7709±1049	12088±2244*	12245±1716*	13906±2385*	4849±394	11060±2295^†^	12040±2132^†^	12565±1091^†^
dP/dtmin (mmHg/s)	−4002±475	−4077±572	−5724±912	−5252±395	−4411±561	−3982±813	−4005±460	−4038±844
SW (mmHg/ml)	10941±1492	12886±1162	12004±2505	13998±637	10020±1456	10620±1937	9776±1553	10237±2322
SV/BW (µl)	1.28±0.38	1.58±0.19	1.13±0.27	0.89±0.20	0.60±0.04*	0.89±0.16^†^	0.98±0.10^†^	0.93±0.08^†^

*P<0.05 vs. NDV, ^†^ *P*<0.05 vs. HFDV.

LV function data at the end of reperfusion are shown in Table 5. At the end of reperfusion, LV function was not different among ND groups. In HFD groups, the ESP and SV/BW was decreased and the EDP was increased in the HFDV group (p<0.05 vs. NDV). All treatments reduced the EDP and increased the ESP and SV/BW in HFD rats (p<0.05 vs. HFDV, Table 5).

**Table 5:** Effects of vildagliptin, metformin, and combination of vildagliptin and metformin on cardiac function at the end of Reperfusion (n = 11/group).

Cardiac function	NDV	NDVil	NDM	NDC	HFDV	HFDVil	HFDM	HFDC
Heart rate (bpm)	281±23	248±18	286±63	237±25	343±48	252±30	281±31	264±25
ESP (mmHg)	82±3	82±14	86±30	96±8	65±4*	91±18^†^	94±6^†^	97±8^†^
EDP (mmHg)	9±3	8±2	11±3	9±3	23±1*	9±3^†^	9±2^†^	11±1^†^
Pmax (mmHg)	117±8	126±10	109±19	97±9	102±10	116±17	110±5	123±9
Pmin (mmHg)	7±2	8±2	8±3	9±4	10±2	6±2	10±2	9±2
dP/dtmax (mmHg/s)	9216±553	12625±2759	10536±1899	10593±2807	8285±1089	9732±1806	10855±816	10776±1761
dP/dtmin (mmHg/s)	−6621±657	−6715±1673	−6431±651	−6551±1434	−7858±1440	−8777±850	−7945±1243	−7550±684
SW (mmHg/ml)	11823±2519	13943±846	10847±743	11095±1073	12374±1219	11775±1270	11434±313	10228±414
SV/BW (µl)	1.02±0.28	1.74±0.26	0.92±0.12	1.00±0.13	0.67±0.02*	1.04±0.18^†^	0.97±0.07^†^	0.94±0.18^†^

*P<0.05 vs. NDV, ^†^ *P*<0.05 vs. HFDV.

### Effects of metformin and vildagliptin on the infarct size

**Figure 3:**
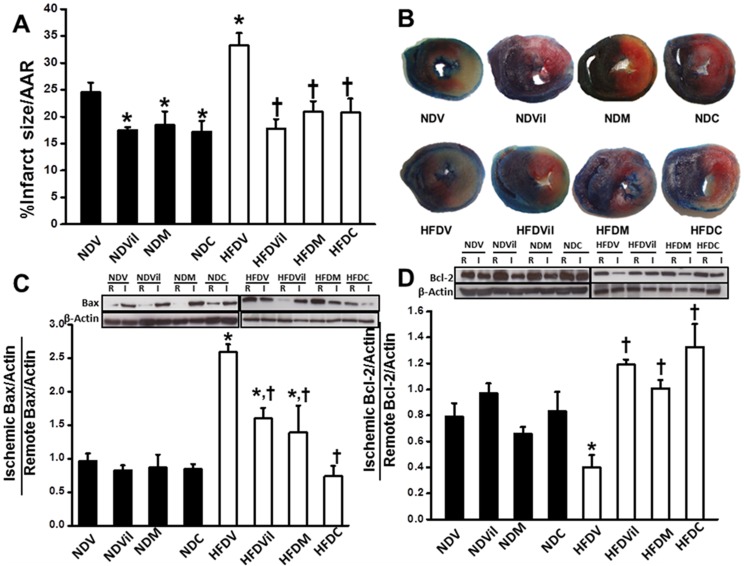
Effects of vildagliptin, metformin, and combined drugs on infarct size, Bax, and Bcl-2 expression. (A) Infarct size was increased in HFDV, and all treatments reduced infarct size in both ND and HFD rats. *p<0.05 vs. NDV, ^†^p<0.01 vs. HFDV, n = 6/group. (B) Representative images showing TTC staining for infarct size determination. (C) Bax expression was increased in HFDV, and all treatments reduced Bax expression in HFD rats. *p<0.05 vs. NDV, ^†^p<0.05 vs. HFDV, n = 5/group. (D) Bcl-2 expression was decreased in HFDV, and all treatments increased Bcl-2 expression in HFD rats. *p<0.05 vs. NDV, ^†^p<0.05 vs. HFDV, n = 5/group.

In this study, the area at risk (AAR) was not different among all groups. We found that the infarct size/AAR was increased in the HFDV group, compared with the NDV group (Figure 3A–B). All treatments reduced the infarct size in both ND and HFD rats (Figure 3A–B). The western blot analysis revealed that the Bax expression was not different among groups of ND rats (Figure 3C). However, the Bax expression was increased in the HFDV rats, compared with the NDV group. In HFD rats, vildagliptin and metformin reduced the Bax expression, compared with the HFDV group. Moreover, Bax expression was markedly decreased in HFD rats treated with combined drugs, compared with the HFDV group (Figure 3C). The Bcl-2 expression was not different among ND groups (Figure 3D). The Bcl-2 expression was decreased in the HFDV rats, compared with the NDV group, and all treatments increased Bcl-2 expression in HFD rats (Figure 3D).

### Effects of metformin and vildagliptin on cardiac mitochondrial function and morphology

**Figure 4:**
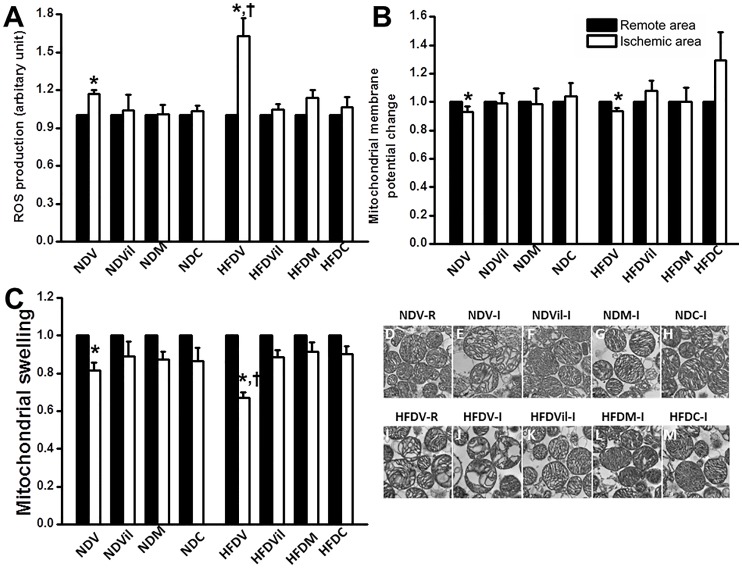
Effects of vildagliptin, metformin, and combined drugs on cardiac mitochondrial function and morphology. (A) Mitochondrial ROS production was increased in ischemic area of NDV and HFDV, Mitochondrial ROS production was higher in HFDV than NDV, and all treatments reduced mitochondrial ROS production. *p<0.05 vs. remote area, ^†^p<0.05 vs. ischemic area of NDV, n = 5/group. (B) Mitochondrial membrane depolarization was found in ischemia area of NDV and HFDV, all treatment prevented mitochondrial membrane depolarization. *p<0.05 vs. remote area of NDV, n = 5/group. (C) An absorbance was decreased in ischemic area of NDV and HFDV. The reduction of absorbance was greater in HFDV than NDV, and all treatments prevented mitochondrial swelling, *p<0.05 vs. remote area of NDV, n = 5/group. (D–M) Cardiac mitochondrial morphology

Our data demonstrated that mitochondrial ROS production was increased in the ischemic area of both NDV and HFDV groups, compared to the remote (non-ischemic) area (Figure 4A). Moreover, the ROS level in the ischemic area was also significantly higher in the HFDV rats, compared to the NDV rats. All treatments reduced mitochondrial ROS production in the ischemic area of HFD rats (Figure 4A).For mitochondrial membrane potential change, the red/green fluorescent intensity ratio was decreased in the ischemic area of both NDV and HFDV groups, compared with their remote areas, indicating mitochondrial membrane depolarization (Figure 4B). All treatments could attenuate mitochondrial depolarization (Figure 4B). For mitochondrial swelling assessment, we found that the absorbance was decreased in the ischemic area of both NDV and HFDV groups, compared with their remote areas, indicating mitochondrial swelling (Figure 4C). Moreover, mitochondrial swelling was significantly greater in the HFDV rats, compared to the NDV rats. All treatments increased the absorbance in both ND and HFD rats (Figure 4C).

Representative images of cardiac mitochondria from the transmission electron microscopy showed the unfolded cristae which is an indicator of mitochondrial swelling. Compared to the intact cardiac mitochondria in the remote area of the NDV rat (Figure 4D), we found that unfolded cristae were mostly found in the ischemic area of the NDV (Figure 4E) and HFDV rats (Figure 4J). Moreover, cardiac mitochondria in the remote area of the HFDV rat (Figure 4I) also had unfolded cristae compared to that in the NDV group, indicating mitochondrial swelling. The marked unfolded cristae were found in the ischemic area of HFDV. All treatments prevented unfolded cristae in both ND (Figure 4F–H) and HFD rats (Figure 4K–M).

### Effects of metformin and vildagliptin on intracellular calcium transient

**Figure 5:**
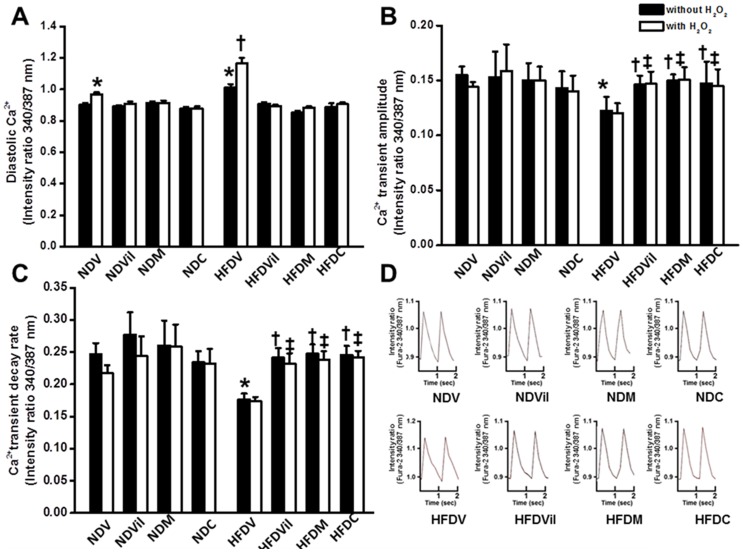
Effects of vildagliptin, metformin, and combined drugs on intracellular calcium regulation. (A) Intracellular diastolic calcium was increased in HFDV. All treatments reduced intracellular diastolic calcium in HFD rats. (B) Calcium transient amplitude was decreased in HFDV. All treatments improved calcium transient amplitude in HFD rats. (C) Calcium transient decay rate was reduced in HFDV. All treatments increased the calcium transient decay rate in HFD rats. (D) Representative calcium transient tracing for calcium transient parameters determination. *p<0.05 vs. baseline NDV, ^†^p<0.01 vs. baseline HFDV, ^‡^p<0.05 vs. HFDV with H_2_O_2_ n = 8 cells/group

Our results demonstrated that the intracellular diastolic calcium level was increased in HFDV hearts, compared with the NDV group (Figure 5A). After treated with H_2_O_2_, intracellular diastolic calcium level was increased in both NDV and HFDV groups, compared with their control. We found that all treatments prevented a change in the intracellular diastolic calcium under oxidative stress condition (Figure 5A).

Calcium transient amplitude was significantly decreased in the HFDV group, compared with the NDV group (Figure 5B). After treated with H_2_O_2_, calcium transient amplitude was not different from baseline of its own group for both NDV and HFDV rats. However, cardiomyocytes from HFD rats treated with vildagliptin, metformin, and combined drugs had a significant increase in calcium transient amplitude both at baseline and with the presence of H_2_O_2_ (Figure 5B).

Calcium transient decay rate was significantly decreased in the HFDV cardiomyocytes, compared with the NDV group (Figure 5C). Calcium transient decay rate in the HFDV group was not different from baseline after treated with H_2_O_2_. All treatments significantly increased calcium decay rate in HFD cardiomyocytes both at baseline and with the presence of H_2_O_2_ (Figure 5C). Representatives of signals of intracellular calcium transients from cardiomyocytes from each group are shown in Figure 5D.

## Discussion

Findings from the present study demonstrated that HFD caused obese-insulin resistance, increased oxidative stress level, depressed HRV, and LV dysfunction. Treatment with vildagliptin, metformin, and combined drugs improved insulin resistance, reduced oxidative stress level, improved HRV, and attenuated LV dysfunction in obese-insulin resistant rats prior to I/R. Under I/R condition, all treatments improved LV dysfunction, reduced the infarct size, prevented cardiac mitochondrial dysfunction, decreased Bax and increased Bcl-2 expression, and reduced cardiac oxidative stress level. Furthermore, all treatments improved intracellular calcium transient regulation in the absence and presence of H_2_O_2_ in the obese-insulin resistant rats. However, only combined drugs could reduce the arrhythmia score, mortality rate, delayed the time to the 1^st^ VT/VF onset, and increased Cx-43 phosphorylation.

In this study, rats developed insulin resistance after 12 weeks of HFD consumption. This finding is consistent with previous studies in which long-term HFD consumption could induce hyperinsulinemia and increased HOMA index, indicating insulin resistance, as well as oxidative stress after 12 weeks of HFD consumption [1], [14], [16], [26]. Previous studies have shown the beneficial effects of vildagliptin and metformin on metabolic parameters [14], [27], [28]. Our data showed that vildagliptin and metformin improved insulin resistance and metabolic parameters as well as oxidative stress in obese-insulin resistant rats. Clinical study reported that vildagliptin in addition to metformin reduced fasting plasma insulin level and HOMA-IR in type 2 diabetic patients [13]. Our study showed that all treatment reduced plasma cholesterol levels. However, the levels of cholesterol in this study were lower than that reported in our previous study [14]. Although these differences could be due to different kits and methods used, all drugs could still decrease the cholesterol levels, compared to the control. Moreover, clinical studies as well as studies in diabetic and insulin resistant animal models reported that vildagliptin and metformin could ameliorate oxidative stress levels [14], [29], [30]. In the present study, rats in all treatment groups had reduced plasma MDA level (prior to I/R) and reduced cardiac MDA level (after I/R), suggesting that vildagliptin and metformin improved insulin resistance and exerted an anti-oxidative stress benefit in obese-insulin resistant rats.

HRV is an indicator of cardiac sympathovagal balance [19]. Previous studies demonstrated that cardiac sympathovagal imbalance was observed in long-term HFD fed rats [14], [31]. Consistent with previous studies, we found that the LF/HF ratio was increased after 8 weeks of HFD consumption, indicating cardiac sympathovagal imbalance. Vildagliptin, metformin, and combined drugs improved the HRV by decreasing LF/HF ratio in obese-insulin resistant rats. Since depressed HRV has been reported to be associated with increased oxidative stress such as an increased MDA level [32], the improved HRV in obese-insulin resistant rats by the drugs found in our study could be due to the reduced oxidative stress caused by these drugs.

Previous studies have shown that long-term HFD consumption caused LV dysfunction [14], [31], and that vildagliptin and metformin could improve LV function in obese-insulin resistant rats [14]. Consistent with previous studies, we found that LV ejection fraction and fractional shortening were reduced after 12 weeks of HFD consumption. Vildagliptin and metformin improved the LV ejection fraction and fractional shortening in the present study. Moreover, we also found that these drugs reduced intracellular diastolic calcium, improved intracellular calcium transient amplitude and decay rate in obese-insulin resistant rats. These findings suggested that one of the mechanisms responsible for improved LV function by vildagliptin and metformin could be attributed to their role in the improved intracellular calcium regulation.

In the present study, vildagliptin and metformin reduced the infarct size caused by I/R injury in obese-insulin resistant rats. Chinda and colleagues reported previously that vildagliptin reduced the infarct size in swine hearts with I/R injury [11], and several studies have demonstrated that metformin also reduced the infarct size in both non-diabetic and diabetic animals [33], [34], [35]. Our results further demonstrated that both vildagliptin and metformin also exerted infarct size reduction benefit in the obese-insulin resistant rat model. The mechanism responsible for this benefit of both drugs could partly due to their anti-apoptotic effects as indicated by reduced Bax and increased Bcl-2 expression. Bax is a pro-apoptotic protein, whereas Bcl-2 is an anti-apoptotic protein, both of which are known to regulate apoptosis during I/R. Furthermore, we demonstrated in the present study that both metformin and vildagliptin could attenuate cardiac mitochondrial dysfunction caused by I/R injury. It has been shown previously that both Bax and Bcl-2 are associated with mitochondrial ROS level [36]. Several studies have shown that metformin improved cardiac mitochondrial function and ultrastructure in insulin resistant rats, diabetic rats, and normal rats with I/R injury [14], [37], [38]. In the present study, we demonstrated that vildagliptin and metformin improved cardiac mitochondrial function and ultrastructure in obese-insulin resistant rats. All of these findings suggested that vildagliptin and metformin reduced the infarct sized by attenuating myocardial apoptosis and cardiac mitochondrial dysfunction. The improved cardiac mitochondrial function as well as infarct size reduction could also be responsible for improved LV function during I/R.

Despite the beneficial effects of metformin and vildagliptin as a single therapy in the present study, we found that only vildagliptin and metformin giving as combined therapy reduced fatal arrhythmias as well as delayed time to the first VT/VF onset during I/R in this obses-insulin resistant rats. This antiarrhythmic effect could be due to the fact that only rats treated with combined metformin and vildagliptin had increased Cx43 phosphorylation. Cx 43 is a cardiac gap junction protein, and it has been shown previously that the engraftment of Cx43 expressing cells could protect the heart against the induction of ventricular tachycardia in mice with myocardial infarction model [39]. Cx 43 phosphorylation at serine 368 has been shown to protect against cardiac arrhythmias during I/R [5], [40]. Our data suggested that the increased Cx43 phosphorylation by combined metformin and vildagliptin could be a mechanism responsible for arrhythmia reduction and leading to decrease arrhythmic death from I/R in obese-insulin resistant rats.

## Conclusions

In conclusion, long-term HFD consumption induced obese-insulin resistance, oxidative stress, cardiac autonomic imbalance, and LV dysfunction. Vildagliptin, metformin, and combined drugs attenuated these adverse effects. Under I/R, these drugs prevented LV dysfunction and reduced infarct size, that was associated with decreased mitochondrial function, improved intracellular calcium regulation, increased anti-apoptotic protein, and reduced pro-apoptotic protein. Only combined metformin and vildagliptin exert better benefits by reduced cardiac arrhythmia score and arrhythmic death during I/R injury in obese-insulin resistant rats by increasing Cx-43 phosphorylation.
